# Low Serum Tryptophan Levels as an Indicator of Global Cognitive Performance in Nondemented Women over 50 Years of Age

**DOI:** 10.1155/2018/8604718

**Published:** 2018-11-21

**Authors:** L. A. Ramos-Chávez, G. Roldán-Roldán, B. García-Juárez, D. González-Esquivel, G. Pérez de la Cruz, B. Pineda, D. Ramírez-Ortega, I. García Muñoz, B. Jiménez Herrera, C. Ríos, S. Gómez-Manzo, J. Marcial-Quino, L. Sánchez Chapul, P. Carrillo Mora, V. Pérez de la Cruz

**Affiliations:** ^1^Laboratorio de Neurobiología de la Conducta, Departamento de Fisiología, Facultad de Medicina, Universidad Nacional Autónoma de México, Mexico; ^2^Departamento de Neuroquímica, Instituto Nacional de Neurología y Neurocirugía Manuel Velasco Suárez, S.S.A., México, DF 14269, Mexico; ^3^División de Neurociencias/Subdivisión de Neurobiología, Instituto Nacional de Rehabilitación S.S.A., México, DF 14389, Mexico; ^4^Instituto Nacional de Estadística y Geografía (INEGI), México, DF 03730, Mexico; ^5^Laboratorio de Neuroinmunología, Instituto Nacional de Neurología y Neurocirugía Manuel Velasco Suárez, S.S.A., México, DF 14269, Mexico; ^6^División de Rehabilitación Geriátrica, Instituto Nacional de Rehabilitación, Ciudad de México, S.S.A. 14389, Mexico; ^7^Laboratorio de Bioquímica Genética, Instituto Nacional de Pediatría, Secretaría de Salud, México City 04530, Mexico; ^8^CONACYT-Instituto Nacional de Pediatría, Secretaría de Salud, Ciudad de México 04530, Mexico; ^9^Laboratorio de Enfermedades Neuromusculares, Instituto Nacional de Rehabilitación, Ciudad de Mexico, S.S.A. 14389, Mexico

## Abstract

Aging is a physiological decline process. The number of older adults is growing around the world; therefore, the incidence of cognitive impairment, dementia, and other diseases related to aging increases. The main cellular factors that converge in the aging process are mitochondrial dysfunction, antioxidant impairment, inflammation, and immune response decline, among others. In this context, these cellular changes have an influence on the kynurenine pathway (KP), the main route of tryptophan (Trp) catabolism. KP metabolites have been involved in the aging process and neurodegenerative diseases. Although there are changes in the metabolite levels with age, at this time, there is no study that has evaluated cognitive decline as a consequence of Trp catabolism fluctuation in aging. The aim of this study was to evaluate the relation between the changes in Trp catabolism and cognitive impairment associated with age through KP metabolites level alterations in women over 50 years of age. Seventy-seven nondemented women over 50 years old were examined with a standardized cognitive screening evaluation in Spanish language (Neuropsi), Beck anxiety inventory (BAI), and the geriatric depression scale (GDS). Also, serum levels of Trp, kynurenine (Kyn), kynurenic acid (KYNA), and 3-hydroykynurenine (3-HK) and the glutathione ratio (GSH/GSSG) were measured. Results showed a negative correlation between age and Trp levels and a positive correlation between age and KYNA/Trp and 3-HK/Trp ratios. The level of cognitive impairment showed a significant positive association with age and with kynurenine pathway activation and a significant negative correlation with Trp levels. The GSH/GSSG ratio correlated positively with Trp levels and negatively with Kyn/Trp and 3-HK/Trp ratios. The depression score correlated negatively with Trp and positively with the 3-HK/Trp ratio. We concluded that KP activation increases with age and it is strongly associated with the level of cognition performance in nondemented women over 50 years of age.

## 1. Introduction

Aging is a time-dependent physiological process that is characterized by a progressive loss of physiological integrity, leading to impairment of functions, and an increased vulnerability to death, affecting all higher organisms [1]. The rising life expectancy leads to higher risk in development of age-related diseases, such as cancer and neurodegenerative diseases [2]. Factors that converge during aging are mitochondrial dysfunction, oxidative stress, decline in antioxidant defense, cellular senescence, stem cell exhaustion, alterations of intercellular communication, genomic instability, epigenetic alterations, deregulated nutrient sensing, and chronic low-grade inflammatory state, among others [3–6].

Some of these factors, such as inflammation and redox state alteration, directly influence the Trp catabolism, which also changes with age. Trp is an essential amino acid, which is metabolized mainly through the kynurenine pathway (KP) (~95%) [7]. Trp can be catabolized by Trp 2,3-dioxygenase (TDO) in the liver and by indoleamine 2,3-dioxygenase (IDO) elsewhere to produce kynurenine (Kyn). Kyn can be a substrate for three enzymes: (1) kynurenine aminotransferases to produce kynurenic acid (KYNA), (2) kynureninase to form anthranilic acid (AA), and (3) kynurenine-3-monooxygenase (KMO) to produce 3-hydroxykynurenine (3-HK), which is further hydrolyzed by kynureninase to 3-hydroxyanthranilic acid (3-HANA). 3-HANA is catabolized as a substrate of 3-hydroxyanthranilate 3,4-dioxygenase which produces an unstable intermediate that rapidly can be converted to quinolinic acid (QUIN) by nonenzymatic cyclization or to produce picolinic acid by the 2-amino-3-carboxymuconate semialdehyde decarboxylase. Finally, quinolinate phosphoribosyl transferase catabolizes QUIN to produce NAD+. KP is controlled mainly by TDO and IDO, which are modulated in different ways. TDO is inducible by glucocorticoids, while IDO is activated by proinflammatory cytokines and superoxide [8–10]. The clinical importance of the KP is due to the fact that metabolites with redox and neuroactive properties as 3-HK, 3-HANA, KYNA, and QUIN are formed through it. QUIN is an agonist of NMDAr, while KYNA is an antagonist of NMDAr and can also inhibit noncompetitively *α*7-nicotinic receptors [11, 12]. It has been observed that brain KYNA level fluctuations impact the cognition [13–21].

There are a few human studies that relate aging with kynurenine pathway components. Pertovaara and coworkers [22] found that the Kyn/Trp ratio is higher in older people than in healthy and younger controls and was able to predict mortality in nonagenarian population. In another study, age was positively associated with kynurenine levels in human serum [23]. It has been shown that picolinic acid concentration in CSF correlates positively with age [24]. Also, KYNA in CSF increased during aging and correlated with high titers of IgG and *β*_2_-microglobulin (markers of immune system activation) [25]. Even with this evidence which suggests that activation of KP occurs during aging and knowing that aging is associated with impairment in cognitive information processing, decline in attention, memory, and other cognitive functions, until now, there have been no studies correlating cognitive performance and KP metabolite levels during aging. The purpose of this study was to determine whether cognitive decline associated with age is related to Trp catabolism fluctuations in nondemented women over 50 years of age.

## 2. Materials and Methods

### 2.1. Chemicals

All chemicals were obtained from Sigma-Aldrich (St. Louis, MO, USA) and J.T.Baker® (Center Valley, PA, USA), unless otherwise mentioned in the text.

### 2.2. Ethical Approval

The protocol, previously approved by the institutional committees (reference no. 114/15), was in agreement with the Declaration of Helsinki and local regulations regarding research on human subjects (Reglamento de la Ley General de Salud en Materia de Investigación para la Salud en México). Written informed consents were obtained from all recruited subjects.

### 2.3. Subject Recruitment Inclusion

The individuals included in the study were 77 adult women, over 50 years old, without evident cognitive impairment, that is, individuals with complete functionality and independent in their basic activities of daily life. No history of neurodegenerative, psychiatric, chronic inflammatory, or autoimmune diseases was present. Also, inclusion criteria required no history of cerebrovascular disease in the previous 6 months and no current use of immunosuppressive or immunomodulatory drugs. Subjects with severe visual or auditory deficit that could affect the cognitive evaluation were excluded. Subjects who fulfilled all the inclusion criteria gave their informed consent to participate in the study.

### 2.4. Cognitive and Emotional Evaluations

For cognitive status evaluation in subjects, a standardized and validated neuropsychological test battery, in adult population, was used, which also allowed to weight the effect of scholarship and age of subjects (Neuropsychological Assessment in Spanish language, *Neuropsi*) [26]. This evaluation has reference standards made in the Mexican population being able to identify individuals with normal performance or with different levels of cognitive impairment as mild, moderate, and severe. Likewise, the Beck anxiety inventory (BAI) and the geriatric depression scale (GDS) were applied, in order to rule out that the cognitive alterations were caused by some emotional state disturbance. All evaluations were performed immediately before the peripheral blood collection.

### 2.5. Sample Blood Collection

Blood samples (5 ml) were collected by vein puncture and allowed to clot, and serum was obtained by centrifugation at 2500 rpm for 20 minutes and stored at −70°C until analysis.

### 2.6. Serum GSH and GSSG Determinations

Serum reduced glutathione (GSH) and oxidized GSH concentrations were determined using a fluorometric method reported by Senft et al. [27] and adapted by Ramos-Chavez et al. [28]. The method is based in the GSH reaction with o-phthaldialdehyde (OPA) to form a highly stable and fluorescent isoindole derivative. Briefly, 50 *μ*l of serum sample was treated with 150 *μ*l of 5% (*w*/*v*) metaphosphoric acid and vigorously mixed. Then, tubes were placed on ice for 15 minutes and centrifuged at 14,000 rpm for 20 minutes at 4°C. 5 *μ*l of supernatant was used for GSH and 30 *μ*l for GSSG determination. For GSSG determination, the first step was to inhibit GSH isoindole derivation using N-ethylmaleimide; subsequently, GSSG is reduced to GSH by dithionite treatment and then derivation with OPA to obtain the isoindole. The fluorescence was measured at 370 nm excitation and 420 nm of emission (*FLx800* Multimode Lector BioTex, Houston, Texas, USA). Calibration curves were built for GSH and GSSG, and the concentrations were obtained by interpolation in the standard curve. The results are expressed as *μ*mol/l.

### 2.7. Kynurenines Determination

Kynurenines were measured by an HPLC method with fluorescence detection for KYNA, Trp, and Kyn, while 3-HK was determined using electrochemical detection [29–32]. The equipment used was a PerkinElmer chromatograph (PerkinElmer, Waltham, MA, USA) coupled to a variable wavelength UV detector (PDA Plus Detector Flexar), a fluorescence detector (model S200), an electrochemical detector (CC-5E LC-4C Amperometric Detector), an automatic delivery pump (Flexar binary LC pump), and an autosampler injector (Flexar LC autosampler). 200 *μ*l of the serum sample was treated with 200 *μ*l of 6% perchloric acid, centrifugated at 14,000 rpm and 4°C. The supernatant was stored at −70°C until analysis.

#### 2.7.1. KYNA and Kyn Determinations

20 *μ*l of the serum supernatant sample or standard solution was injected onto an Eclipse XDB-C18 reverse phase column (5 *μ*m, 4.6 × 150 mm, Agilent, Santa Clara, CA, USA) and isocratically eluted with a mobile phase consisting of 50 mM of sodium acetate, 250 mM of zinc acetate, and 3% of acetonitrile, pH adjusted to 6.2 with glacial acetic acid, at a flow rate of 1 ml/min; Kyn was eluted with the same mobile phase but without acetonitrile. Both metabolites were detected by fluorescence, KYNA at excitation wavelength of 344 nm and emission wavelength of 398 nm and Kyn at excitation wavelength of 368 nm and emission wavelength of 480 nm. The retention time for KYNA was ~7 min and for Kyn was ~10 min.

#### 2.7.2. Trp Analysis

Trp levels were determined using a ZORBAX Eclipse AAA column (3.5 *μ*m, 4.6 × 150 mm, Agilent, Santa Clara, CA, USA) and isocratically eluted with a mobile phase containing 100 mM of zinc acetate and 3% of acetonitrile (pH adjusted to 4.2 with glacial acetic acid) at a flow rate of 1 ml/min. 20 *μ*l of biological sample or standard solutions was injected for Trp determination. Trp was detected by fluorescence (excitation wavelength: 254 nm and emission wavelength: 404 nm). The retention time of Trp was ~5 min.

#### 2.7.3. 3HK Measurement

The 3HK was determined using an electrochemical method described by Heyes and Quearry et al. [29]. Briefly, 3HK was eluted at a constant flow rate of 0.6 ml/min with a mobile phase containing 9% of triethylamine, 0.59% phosphoric acid, 0.27 mM EDTA, and 8.9 mM heptane sulfonic acid; 40 *μ*l of the sample or standard was injected onto an Adsorbosphere Catecholamine C18 reverse phase column (3 *μ*m, 4.6 mm × 100 mm, Fisher Scientific, Hampton, Nuevo Hampshire, USA). The retention time was ~11 min.

### 2.8. Statistical Analysis

Correlation between each pair of the following variables was assessed with Spearman's rho coefficient: age, cognition, Trp, Kyn/Trp, KYNA/Trp, 3-HK/Trp, depression score, anxiety, and GSH/GSSG. The Mann-Whitney test and *T*-test were used to compare distributions associated with normal (undetected) and detected cognitive impairment groups; the *T*-test was obtained using the logarithmic scale. Finally, a logistic regression model was adjusted considering age and Trp levels in the logarithmic scale as covariables, where the dependent variable corresponds to the binary variable associated with cognitive impairment (1 = normal and 0 = CI). The logistic regression model obtained was the following:

(1) $$\begin{array}{c} \log\left(\frac{p}{1-p}\right)=-31.9+7.7^{*}\times \log\left(age\right)-0.83^{**}\times \log\left(Trp\right). \end{array}$$

## 3. Results

Demographic and clinical characteristics of the participants are shown in Table 1. The average age was 71.9 years (SD: 11.8), the average number of years of education was 8.4 (SD: 5.0), and only 26% of them had education over 9 years. 77% of them had between 1 and 3 comorbidities (e.g., hypertension, diabetes, dyslipidemia, osteoarthritis, and obesity). Regarding their cognitive performance, 70% of them obtained a normal evaluation, and 30% (*n* = 23) presented some degree of cognitive impairment (CI). 33% found significant symptoms of depression and 53% with some symptoms of anxiety. Descriptive statistics of analytes is shown in Table 2.

**Figure 1:**
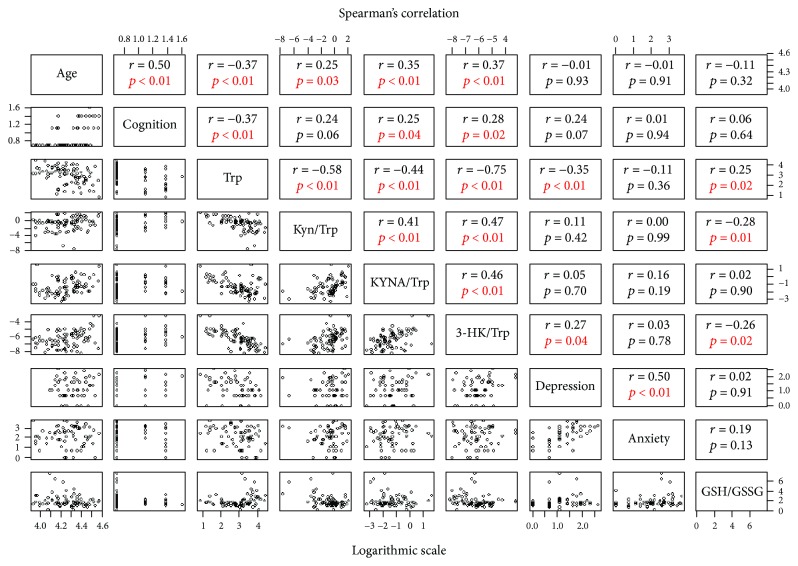
The lower triangular matrix contains the scatterplot for each pair of variables in the logarithmic scale. The upper triangular matrix contains Spearman's rank correlation coefficient and its associated *p* value. In this case, the variable cognition corresponds to an ordinal variable with four categories depending on the level of cognitive impairment: normal, mild, moderate, and severe.

Pairwise correlations among age, cognition level, Trp levels, and the Kyn/Trp, KYNA/Trp, and 3-HK/Trp ratios as well as depression, anxiety, and GSH/GSSG ratio are showed in Figure 1. As expected, age correlated positively with the cognitive impairment level (four ordinal categories were used: normal, mild, moderate, and severe). Trp levels (pmoles/*μ*l) correlated negatively with age, while Kyn (pmoles/*μ*l)/Trp, KYNA (fmoles/*μ*l)/Trp, and 3-HK (pmoles/*μ*l)/Trp ratios correlated positively with age. Also, the cognitive impairment level was associated negatively with Trp levels and positively with KYNA/Trp and 3-HK/Trp ratios. As it has been described by other groups [23], the depression score correlates positively with anxiety and negatively with the levels of Trp, as was observed in this study. We also found a positive correlation between the depression score and 3-HK/Trp ratio. The GSH/GSSG ratio was determined as a redox status marker and correlated positively with Trp and negatively with Kyn/Trp and 3-HK/Trp ratios.

To extend the analysis on the association between Trp catabolism and cognitive impairment, only two groups of subjects were considered. The first one consisted of those subjects who did not present with cognitive impairment, the second, those that presented some level of cognitive impairment. The results of the statistical tests comparing the distributions of these two groups for the serum levels of Trp, Kyn/Trp, KYNA/Trp, and 3-HK/Trp are shown in Table 3. It was found that the levels of Trp were significantly different in women with cognitive impairment in comparison with those without any cognitive impairment; the median of the Trp levels in women with cognitive impairment was around half the median value found of those in women without cognitive impairment (Table 3). Also, Kyn/Trp, KYNA/Trp, and 3-HK/Trp serum ratios were significantly different between women with cognitive impairment and those without any cognitive impairment.

**Figure 2:**
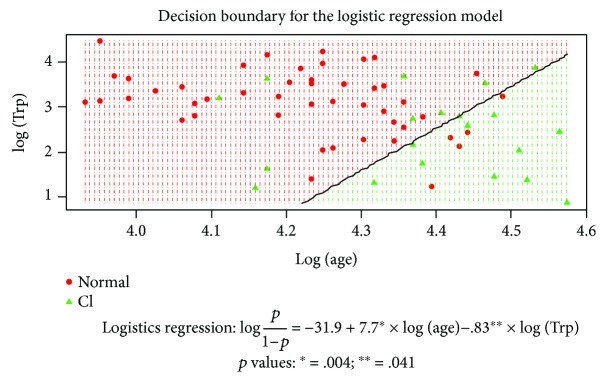
Scatterplot for age and Trp in the logarithmic scale distinguishing between the status of cognitive impairment: detected and normal. The adjusted logistic regression considering these two variables where the dependent variable corresponds to the binary variable associated with cognitive impairment (1: detected and 0: normal) is also shown together with the corresponding *p* values for the coefficients in the logistic regression model.

Moreover, to study the correlation between the presence of cognitive impairment and Trp levels, a logistic regression model including age as a covariable was adjusted; as was mentioned before, there was a significant correlation between age and cognitive impairment. The results show that both covariables, age and Trp levels, are significant in the model, and for a given age, it means that at lower Trp levels, there is a greater probability of observing cognitive impairment.  Figure 2 shows the decision boundary for the adjusted logistic regression.

## 4. Discussion

According to our knowledge, this is the first time that Trp metabolites have been correlated with cognitive performance during normal aging in women. Our results suggest an overactivation of the KP during aging since we found a negative correlation between age and Trp levels and a positive influence of age on Kyn/Trp, KYNA/Trp, and 3-HK/Trp ratios; which is supported by the fact that the serum Kyn/Trp ratio is a measure of the beginning of KP activity [33, 34]. These data are consistent with previous studies in which low plasma, serum, and CSF Trp levels and high values of the Kyn/Trp ratio were also observed in elderly people [22, 23, 33, 35–39]. These alterations on the Kyn/Trp ratio may be due to enhanced activity of IDO and/or TDO. Although the major site of Trp conversion into kynurenine is the liver via TDO, it has been shown that liver TDO activity, both holoenzyme and apoenzyme, decreases significantly with age in rats [40], so that if we consider that during aging there is an increased concentration of inflammatory markers, the Kyn/Trp ratio could reflect mainly IDO activity [41]. Supporting this idea, it has been previously described that IL-6 correlates positively with Kyn and the Kyn/Trp ratio in serum of older people [4], suggesting that IDO activity increases with aging [37]. In addition, it has been found that the Kyn/Trp ratio and neopterin concentration (marker associated with inflammation and oxidative stress) were significantly correlated with increased age, while Trp correlated negatively with age in CSF samples from women [33].

Additionally, our results indicated a strong association between KP activation and redox status. The ratio of GSH and GSSG (GSH/GSSG) has been noted as an index of oxidative stress. Specifically, in this study, the GSH/GSSG ratio correlated negatively with KP activation, and as was mentioned before, some metabolites produced through KP have neuroactive and redox properties [10]. Kyn and KYNA have shown antioxidant properties [32, 42], while 3-HK and 3-HANA can also be scavengers in a concentration-dependent way and after their interaction with ROS leads to more toxic compounds inducing cellular death [43–45]. An important point is that GSH levels can be reduced because they can produce an adduct with the 3-HK glucoside, which, at the same time, is a product of 3-HK deamination [46, 47]. Keeping this in mind, the low GSH/GSSG ratio found in this study could be related to the high levels of redox kynurenines.

In this study, the Kyn/Trp, KYNA/Trp, and 3-HK/Trp ratios that are associated with age reflect a greater amount of KP metabolites in the circulation. However, just Trp, Kyn, and 3-HK can cross the blood-brain barrier, either through simple diffusion across the vascular membranes or as a result of active transport via the large neutral amino acid transporter [48–51]. Recently, Hestad and coworkers showed that serum Kyn levels correlated highly with CSF Kyn levels [23]. The alterations of blood KP metabolites can produce significant secondary changes in the levels of kynurenine metabolites in the CNS and consequently impact processes such as cognition, by altering the degree of activation or blockade of NMDAr as well as the *α*-7 nicotinic receptor [52, 53].

In this context, the 3-HK/Trp ratio is consistent with the increase in downstream metabolites such as 3-HANA, PIC, and QUIN found in human CSF, with age. In rats the increase of QUIN with age has also been described [24, 33, 54]. Moreover, Trp and Kyn that really cross the blood-brain barrier, can produce KYNA by KATs, considered the canonical way. However, there are other mechanisms by which KYNA is produced and that involve the interaction of D- and L-isomers of Trp and Kyn with reactive oxygen species (ROS) [32, 55, 56], which as we know, is an important factor during aging. Fluctuation in KYNA levels leads to behavioral and cognitive changes during aging [25]. According to data obtained in this study, Trp catabolism through KP and the level of cognitive impairment are associated with aging in women over 50 years of age. Experimental studies have shown that increased levels of Kyn are linked with deficits of spatial working memory [17, 57]. The deletion of the major KAT isoenzyme, KAT-II, results in a substantial decrease in the extracellular concentration of KYNA, improving cognitive performance in a range of behavioral tasks which include exploration, object recognition, and passive avoidance learning [20]. Also, it has been demonstrated that Trp depletion affects a variety of cognitive processes in healthy individuals, such as memory and learning skills and long-term memory consolidation, which can be associated with the bioactive kynurenines such as KYNA and QUIN [58, 59].

A strong relationship between KP activation and cognitive impairment is also observed in Alzheimer's disease, which is an age-related neurodegenerative disease. Widner and coworkers found decreased serum Trp levels and increased serum kynurenine levels, and these changes correlated with the level of cognitive decline in Alzheimer's disease patients [60, 61]. Also, plasma Trp concentrations were found to be lower in HIV+ compared with HIV− individuals, and a higher plasma Kyn/Trp ratio was associated with cognitive impairment and major depressive disorder in the overall HIV+ group [62]. Another study found positive correlations between cognitive function tests and lower plasma KYNA levels, and inverse correlations between these tests and increased QUIN levels in Alzheimer's patients [63]. Plasma and the CSF Kyn/Trp ratio were correlated with risk of dementia in Alzheimer's patients [63–65]. Interestingly, it has also been observed that increased KP activation and changes in the levels of kynurenine metabolites correlate negatively with cognitive performance in patients undergoing cardiac surgery, which suggest that Trp catabolism can be a biomarker of non-age-related cognitive impairment [66].

Noteworthy to mention is that in this study, Trp levels correlated negatively with the depression score and positively with the 3-HK/Trp ratio and anxiety in women over 50 years of age. These results suggest the KP activation in depression, which may induce a transient reduction in serotonin synthesis, which may also be associated with depression [67–71]. In our study, we did not take into account serotonin production, considering that the serotonin synthesized in the periphery is unable to cross the blood-brain barrier, whereas the production of brain serotonin is dependent on the amount of circulating Trp [72]. Low Trp intake has been assumed to cause lower brain serotonin levels and to be an important risk factor involved in the onset and course of a variety of affective disorders, including depression [73]. In this context, Suga and coworkers showed an inverse association between Trp intake and depressive symptoms in young women participants (mean age around 18 years old), which suggests that the adequate intake is necessary to prevent depression [74]. A recent study observed that the increased Trp catabolism related to peripheral inflammation is accompanied by marked elevation in brain kynurenine and QUIN levels, and these alterations correlated with depressive symptoms in patients with hepatitis C [75].

Interestingly, our analysis suggests elements to establish that circulating Trp levels are a predictive biomarker and could be used to distinguish women over 50 years of age with some degree of cognitive impairment. However, it is important to take into account that this study was performed in women over 50 years of age; it is therefore necessary to confirm these results in a wider age range and to determine whether this effect is also present in men, in order to establish whether Trp levels are predictive for cognitive impairment in the general population.

## 5. Conclusion

The identification of novel biomarkers associated with the cognitive impairment that occurs during aging could provide key biological insights to identify an adequate intervention. This study confirms a close relationship between age, the Trp catabolism through KP activation, and cognitive impairment. However, based on our logistic regression model, given the age, Trp levels were the only significant predictor among the KP metabolites. Then, Trp levels can be considered as a useful indicator of cognitive impairment in women over 50 years of age, and these results afford a basis for further investigation in order to design future intervention strategies focused on prevention and treatment of cognitive impairment.

## Figures and Tables

**Table 1:** Clinical and demographic features of all individuals (*n* = 77).

Age (years)	Mean ± SD	71.92 ± 11.84
	Min, Max	51.00–97.00

Education	Mean ± SD	8.48 ± 5.02
	0–4 years	11 (14%)
	5–9 years	46 (60%)
	10–24 years	20 (26%)

Civil status	Married	30 (39%)
	Single	19 (25%)
	Widow	28 (36%)

Occupation	Paid employment	47 (61%)
	Vacant/retired	30 (39%)

Comorbidities	0–3	59 (77%)
	4–6	16 (20%)
	7–9	2 (3%)

Cognition profile	Mean ± SD	85.86 ± 20.24
	Min, Max	23.00 ± 119.00
	Normal	54 (70%)
	Mild impairment	10 (13%)
	Moderate impairment	11 (14%)
	Severe impairment	2 (3%)

GDS (depression), *n* = 63	Mean ± SD	3.44 ± 2.60
	Min, Max	0.0 ± 10.00
	Without depression symptoms	42 (67%)
	With depression symptoms	21 (33%)

BAI (anxiety)	Mean ± SD	12.52 ± 10.98
	Min, Max	0.0–42.00
	Minimal	36 (47%)
	Mild	21 (27%)
	Moderate	14 (18%)
	Severe	6 (8%)

GDS: geriatric depression scale; BAI: Beck anxiety inventory.

**Table 2:** Descriptive statistics of GSH, GSSG, and KP metabolites for each group (normal and detected cognitive impairment (CI)).

	Min	Percentile 25	Median	Mean	Percentile 75	Max	*n*
GSH (*μ*mol/l)							
Total	53.760	151.030	196.860	209.920	236.740	766.000	79
Normal	53.760	148.710	187.210	206.580	220.590	766.000	48
CI	129.700	165.100	218.800	213.900	253.300	340.400	18
GSSG (*μ*mol/l)							
Total	0.090	12.650	40.180	39.700	62.800	118.780	79
Normal	0.090	14.480	41.440	41.750	63.470	118.780	48
CI	7.410	19.430	40.720	43.260	67.950	85.270	18
Trp (pmoles/*μ*l)							
Total	2.380	11.450	22.740	26.360	37.290	87.600	82
Normal	3.401	15.802	23.370	28.208	35.173	87.599	48
CI	2.380	5.017	11.472	15.384	17.506	47.816	21
KYNA (fmoles/*μ*l)							
Total	0.832	2.708	4.525	10.534	6.662	209.894	79
Normal	0.832	2.514	4.465	13.325	7.191	209.894	47
CI	1.694	2.768	3.731	4.595	4.908	15.561	19
3-HK (pmoles/*μ*l)							
Total	0.000	0.025	0.036	0.046	0.053	0.376	82
Normal	0.000	0.025	0.041	0.050	0.057	0.376	48
CI	0.006	0.026	0.036	0.045	0.052	0.155	21
L-Kyn (pmoles/*μ*l)							
Total	0.000	3.478	12.513	20.305	22.417	283.361	80
Normal	0.015	5.636	14.697	20.158	22.820	224.934	47
CI	2.174	6.396	12.513	14.127	17.542	39.094	20

**Table 3:** Descriptive statistics of Trp levels and Kyn/Trp, KYNA/Trp, and 3-HK/Trp ratios for each group (normal and detected cognitive impairment (CI)).

	Trp		Kyn/Trp		KYNA/Trp		3-HK/Trp	
	Normal	CI	Normal	CI	Normal	CI	Normal	CI
*Descriptive statistics*								
Percentile 25	15.802	5.017	0.198	0.359	0.117	0.236	0.001	0.001
Median	23.370	11.472	0.579	0.856	0.191	0.459	0.002	0.003
Percentile 75	35.173	17.506	1.045	3.094	0.352	0.686	0.003	0.005
Mean	28.208	15.384	0.915	2.120	0.454	0.661	0.003	0.007
*n*	48	21	47	18	46	19	47	21
*p values*								
Mann-Whitney test	0.0015		0.0700		0.0280		0.0262	
*T*-test	0.0017		0.0350		0.0306		0.0189	

## Data Availability

The cognitive and biochemistry data used to support the findings of this study are available with the corresponding author upon request.
